# Tumor Invasion Optimization by Mesenchymal-Amoeboid Heterogeneity

**DOI:** 10.1038/srep10622

**Published:** 2015-05-27

**Authors:** Inbal Hecht, Yasmin Bar-El, Frederic Balmer, Sari Natan, Ilan Tsarfaty, Frank Schweitzer, Eshel Ben-Jacob

**Affiliations:** 1 School of Physics and Astronomy, The Raymond and Beverly Sackler Faculty of Exact Sciences, Tel Aviv University, Tel Aviv 6997801, Israel; 2 Chair of Systems Design, ETH (Eidgenoessische Technische Hochschule) Zurich, Weinbergstrasse 58, 8092 Zurich, Switzerland; 3 Department of Clinical Microbiology, Faculty of Medicine, Tel Aviv University, Tel Aviv 6997801, Israel; 4 Center for Theoretical Biological Physics, Rice University, Houston, TX 77005-1827, USA

## Abstract

Metastasizing tumor cells migrate through the surrounding tissue and extracellular matrix toward the blood vessels, in order to colonize distant organs. They typically move in a dense environment, filled with other cells. In this work we study cooperative effects between neighboring cells of different types, migrating in a maze-like environment with directional cue. Using a computerized model, we measure the percentage of cells that arrive to the defined target, for different mesenchymal/amoeboid ratios. Wall degradation of mesenchymal cells, as well as motility of both types of cells, are coupled to metabolic energy-like resource level. We find that indirect cooperation emerges in mid-level energy, as mesenchymal cells create paths that are used by amoeboids. Therefore, we expect to see a small population of mesenchymals kept in a mostly-amoeboid population. We also study different forms of direct interaction between the cells, and show that energy-dependent interaction strength is optimal for the migration of both mesenchymals and amoeboids. The obtained characteristics of cellular cluster size are in agreement with experimental results. We therefore predict that hybrid states, e.g. epithelial-mesenchymal, should be utilized as a stress-response mechanism.

Despite the impressive advances in cancer research and therapy, cancer metastases are still largely incurable and are tightly related to patient death^1^. Formation of secondary lesions (metastases) involves detachment of cancerous cells from the primary tumor, migration towards the blood vessels and relocation to distant organs^2^,^3^. This challenging expedition demands advanced navigation strategies to deal with the complex and frequently-changing environment of the surrounding tissue and extra-cellular matrix (ECM).

Upon detachment from the primary tumor, the cells can adopt one of several motility modes. The main modes are the “path generating” mesenchymal mode, and the “path finding” amoeboid mode. Mesenchymal cells utilize proteolysis to degrade the surrounding ECM and generate new paths for them to move through. Some hybrid phenotypes, exhibiting both epithelial and mesenchymal attributes, have also been reported and studied^4^,^5^. Therefore, mesenchymal characteristics are not limited to individually migrating cells and can be also used by collectively migrating cancer cells^6^.

Amoeboid cells are more flexible and can squeeze in small gaps in order to find their way, but are not able to degrade the ECM or generate new paths. Nonetheless, amoeboid motility can be as effective as mesenchymal motility. In the presence of proteolytic inhibitors, mesenchymal cells spontaneously switch to the amoeboid form, and thus maintain their ability to migrate^7^,^8^,^9^. Amoeboid motility was also found to be preferred in situations of metabolic stress, such as hypoxia, which may imply that the amoeboid motility mode is less energy-consuming or more efficient compared to the mesenchymal mode. In our previous work^10^ we have shown that amoeboid motility, with its special ability to quickly and efficiently adjust the cell shape, is highly competent for navigation in complex environments and bypassing obstacle. To assess the navigational ability of amoeboid cells we used different types of obstacles, from small, local barriers to longer walls (maze-like geometry). In this study we continue to use the maze as a representation of the complex ECM environment.

Other forms of cell migration include multi-cellular amoeboid migration, clusters, cellular streaming (strands) and cell sheets^6^,^11^,^12^,^13^. The mechanisms that govern the transition from one form to another include mechanical forces, signaling and environmental conditions^4^,^5^,^7^, but are not yet fully understood.

In this work we focus on the invasion and migration of cells that have detached from the primary tumor, i.e. have gone through Epithelial-to-Mesenchymal Transition (EMT) or Epithelial-to-Amoeboid Transition (EAT). To distinguish between stromal mesenchymal cells, and epithelial cells that transformed and obtained mesenchymal characteristics, the latter are usually termed “EMT cells”. Amoeboid cells of epithelial origin are similarly termed “EAT cells”.

In the last decade, the reductionist view of cancer as a collection of individually proliferating cells has been gradually replaced with the view of cancer as a complex network of interacting cells^14^,^15^,^16^. Cancer cells, both in the primary tumor and metastasizing, communicate via chemical signals that affect proliferation, differentiation and migration^17^ as well as mechanical signals, that affect cellular morphology and acceleration^18^,^19^,^20^. The study of cancer dissemination should therefore include both the single-cell as well as the multi-cellular levels, in order to genuinely understand the mechanisms that are involved in the metastatic process.

A migrating cancer cell is typically surrounded by a large number of other cells, all moving in the same limited and packed environment. Figure 1a shows an *ex-vivo* microscopy of a breast cancer tumor, in which cell nuclei are shown in blue and the collagen fibers of the ECM are shown in red. In such a dense environment of cells and ECM fibers, each cell is clearly influenced by the other cells. Indirect interaction between the cells is caused by changes in the physical microenvironment made by one cell that affect other cells in the same area^3^. For example, ECM degradation or repatterning made by one cell may improve and enhance the migration of other cells. Experiments have shown that single cells may act as “forerunners” that generate a path for the benefit of follower cells^12^,^21^. Thus, mesenchymal migration ability may exhibit a non-linear dependence on the number of EMT cells, due to an accumulative effect of ECM degradation. Moreover, a small fraction of EMT cells may influence the migration pattern and invasion ability of the entire, mostly EAT, population.

In our previous work we looked at the motion of a single cell with full shape dynamics^10^ or a single agent and measured the success rate in the maze. In this work we wanted to look into “synergism” effects, namely the influence of many cells moving in the same area on their combined success rates. In live cells or organisms, it is often found that mechanisms of decision making of an individual are tuned to benefit the entire population and not necessarily the individual itself. Some examples for this phenomenon include bees foraging patterns^22^ and transition to competence in bacteria^23^. Actions of a single cell that benefit other cells can therefore be regarded as effective cooperation, although no direct collaboration between the cells is evident. Such cooperation can inexplicitly emerge from allegedly independent single-cell behavior, by the nature of their decision making mechanisms.

In this work we study how single-cell motility can enhance the metastatic ability of the entire cancer cell population. Specifically, we simulate a fixed number of cells, both EMT and EAT, in a maze, and measure their success in crossing the maze within a limited time, for different ratios of the EMT and EAT populations and different environmental conditions. As we look at the population level, we use here a coarse grained model of cell motion without detailed shape dynamics. Therefore, the cells are represented by self-propelled agents that move and invade their surroundings similarly to actual cells visualized in low spatial resolution. Similarly to our previous work and other models of Brownian agents^24^, the agents have the ability to obtain metabolic energy from the environment and use it to actively move and change their environment. It was demonstrated that models of Brownian agents describe real motion at different levels of biological organization, ranging from intracellular vesicles^25^, to cells^26^, bacteria^27^, Daphnia^28^, ants^29^, and swarms^30^.

We find that the presence of a small population of EMT cells can affect the migration of EAT cells in the same environment, if the resource level (termed “energy”) is sufficient. Under metabolic stress, on the other hand, the population does not benefit from the presence of EMT cells and the amoeboid motility mode is beneficial. We also studied possible direct interactions between the cells and found that direct alignment interaction is beneficial for EMT cells under low- and mid-energy conditions, but has a small destructive effect when the energy is high. For amoeboids, the effect of interaction is different. It is slightly beneficial for low energy, but is detrimental for mid- and high-energy conditions, as cell-cell interaction results in cellular aggregation with low invasion rate. We therefore suggest that cell-cell interaction may be regulated as a stress-response mechanism, and present the case of energy-dependent interaction. Our results of cellular clustering and group size are analogous to experimental results of cancer cells, both melanoma and fibrosarcoma, moving in 3D collagen matrix^19^.

## Model

### General description

**Figure 1:**
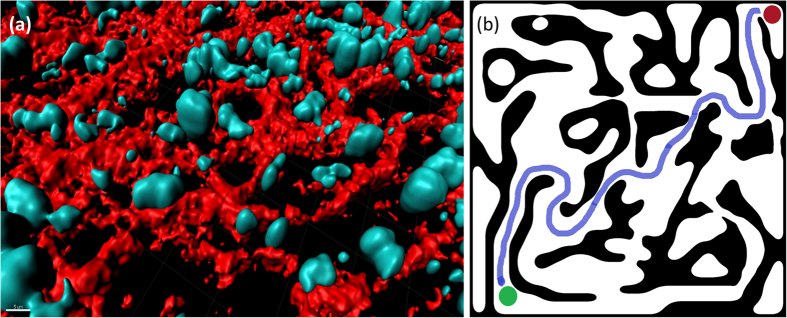
The environment in which invasion takes place. (**a**) Experimental: nuclei of breast cancer cells (blue) and the collagen fibers (red) in a mammary tumor (*ex vivo*). The cells move in a dense mesh of ECM fibers. Scale bar, 5 µm. (**b**) The simulation maze: The white spaces are open, while the black spaces are walls. All agents are initially placed at the starting zone (green circle) and are guided by the chemoattractant gradient to the target zone (red circle). A possible typical trajectory in the maze is shown by the blue curve.

In this work, the cells are represented by self-propelled agents. This is a coarse-grained method that encapsulates cellular dynamics by the overall motility characteristics, without dealing with the detailed dynamics of cellular shape. In our previous study^10^ we used round obstacles as well as a square maze with straight lines. In this work we use a more “natural” form of maze-like geometry (Fig. 1b), with rounded corners and a combination of blocking obstacles and open paths. A possible trajectory in the maze is presented, with the starting and ending points (green and red, respectively). In the Supporting Information Text we present additional results of our simulation with another maze of a different kind. The two mazes presented here have similar average density but different pore size and wall thickness, and we have also tested other mazes with higher and lower density. While the exact success rates depend on the specific maze characteristics, such as pore size and wall thickness, our qualitative findings are robust and are independent of the specific maze: the effective cooperation between mesenchymals and amoeboids and the role of interaction are similar in the different mazes.

Each simulation is run with 50 agents, each of them assigned with a “mesenchymal” or “amoeboid” motility mode, representing EMT and AET cells, respectively. In subsequent description of the model and results the agents are referred to as “mesenchymals” and “amoeboids”, for better readability. The relative sizes of the mesenchymal and amoeboid populations are kept constant throughout the simulation, as we want to assess the role of the indirect interaction between the different cell types. Thus, the motility mode of each agent is permanent, in order to keep the populations’ ratio always constant. The role of mesenchymal-amoeboid and amoeboid-mesenchymal transitions (MAT/AMT respectively) should be studied elsewhere.

Real cells need energy for basic cellular maintenance, migration and proliferation, as well as for protein synthesis and secretion of signaling molecules. The needed energy is obtained by metabolism of sugars and fats that are absorbed from the environment. The overall limited energy of the cell creates an effective competition between the different cellular tasks, which becomes more significant when the cell is under metabolic stress which limits the available energy. In our model, each agent obtains energy in a constant rate, which is a characteristic of the resource level in the environment. The agent then spends its energy on migration and maze-wall degradation, as described below. By varying the energy intake rate we can mimic different situations of environmental conditions and test the single-cell as well as the population performance under different metabolic conditions.

We define a starting point in the maze, where all the agents are initially located, and an ending point, where agents are considered successful. We let the agents move (see details below) and count the number of agents that reached the ending point within the defined simulation time. The detailed force and energy equation are given below.

The agents’ motion in the maze consists of the following steps:

1. Orientation: We assume a directional cue that directs the agents from the starting to the ending point, similarly to a chemoattractant gradient for chemotaxing cells. Importantly, this signal is not limited or affected by the maze walls, so an agent may be directed to a blocking wall. Each agent chooses its direction according to the external cue with a Gaussian noise (see SI Table for parameter details). The directional noise is taken to be larger for amoeboids compared to mesenchymals, according to experimental results showing a high directionality for mesenchymal cells and low directionality for amoeboids^31^. The noise is added every time the cell reorients.
2. Locomotion: the agent moves in the selected direction until it encounters a wall or until re-orientation time.
3. Interaction with walls: When an amoeboid agent encounters a wall, its velocity term that is perpendicular to the wall is zeroed, while the tangent velocity term is unaffected, which results in the agent “sliding” along the wall. When a mesenchymal agent encounters a wall, it may degrade it according to its energetical state (see below).
4. Energy management: During the entire journey the agent obtains energy from the environment and spends it on motility and wall degradation.

### Model equations

Each agent (denoted by the subscript *i*) has the following characteristics: 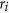, its position; 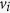, its velocity; 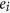, its internal energy; 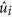, its current direction of propulsion as determined by the orientation step.

#### Motion equations

The motion of an agent is governed by the following Newton’s equations:
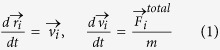
Where the mass m is further assumed to be equal to 1 for all agents.

The total force acting on the agent is the sum of the propulsion and drag forces:
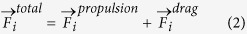
Propulsion only happens when the current stage of agent *i* is locomotion. We identify the orientation/locomotion switch by 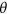, i.e. 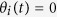 during the orientation stage and 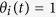 during the locomotion stage. Propulsion is characterized by *η*, the rate of conversion of internal energy into kinetic force, and the unit vector 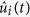, which is the direction of the agent:
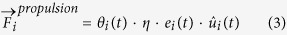


The direction vector 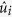 is given by the external pre-defined direction, which in our case is actually the vector pointing to the target point. A Gaussian noise with variance 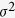 is added to this deterministic direction. The noise distribution width 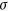 varies between mesenchymal and amoeboid agents: the “path generator” mesenchymals have a lower noise compared to the “path finder” amoeboids (see Supporting Table T1 for the exact values).

The drag force is characterized by a migration-mode dependent coefficient:
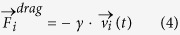
where 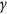 is either 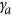 or 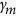 for amoeboid or mesenchymal agents, respectively. Since mesenchymal cells have stronger cell-ECM adhesion, we take 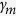 > 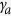 . This distinction stems from the difference in cell-ECM adhesion between amoeboid and mesenchymal cells^9^,^13^,^32^, which results in different effective friction that influences the speed of the two agent types (with the amoeboids having weaker adhesion and larger velocity^33^). All parameter values can be found in the Supporting Table T1.

#### Energy dynamics

As mentioned above, the different cellular tasks consume energy, which is typically obtained from the environment. Energy dynamics is given by:
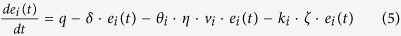
Where *q* is the constant rate of intake of energy by the agent, 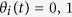 as described above and 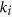 indicates whether the agent is currently able to degrade the ECM (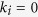 for amoeboids, 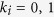 for a mesenchymal cell that is unable/able to degrade, respectively). In this model, the only restriction to ECM degradation is the energy level of the agent, which needs to exceed the degradation energetic cost.

The second term on the right hand side simply expresses the dissipation of internal energy due to, for example, basic cellular maintenance. The third term is the expression of the mechanical power needed to propel the agent. The fourth term describes the use of energy for proteolysis, and the constant 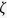 characterizes the energetical cost of maze wall degradation. In our previous work, which focused on single cell invasion, we studied a more detailed model of energy intake as a function of the external resource level and the metabolism rate, including the influence of signaling that affects the metabolism rate. In this work we focus on the population, and therefore embed the metabolic and environmental details into a single parameter *q*.

## Results

### Trajectories and success rate

**Figure 2:**
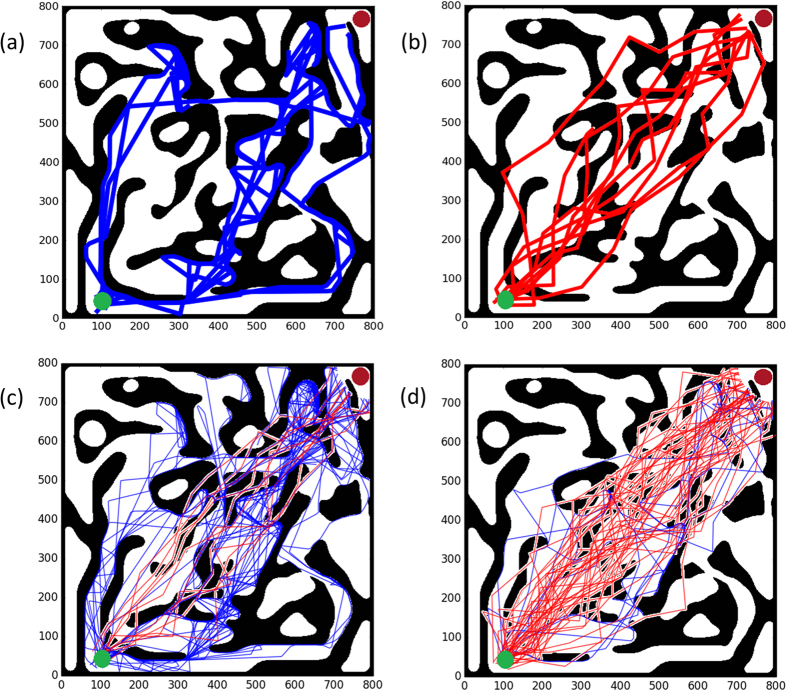
Trajectories. (**a**)–(**b**) Typical trajectories of 10 amoeboid (**a**) and mesenchymal (**b**) agents. (**c**)-(**d**) The trajectories of amoeboids (blue) and mesenchymal (red) cells for q = 1 and two different population distributions: (**a**) 20% mesenchymals and 80% amoeboids; (**b**) 80% mesenchymals and 20% amoeboids.

The total number of agents in the group was kept constant, with changing ratios of mesenchymal and amoeboids. Possible trajectories are shown in Fig. 2, for 10 agents of a single type (Fig. 2a-b) and for 50 agents with 20% or 80% mesenchymals (Fig. 2c-d). When more mesenchymals are present, the amoeboids seem to be less scattered in the maze, and their trajectories are more localized in the central zone, around the diagonal line between the start and end points. Videos of the simulations shown in Fig. 2c-d are shown in the Supporting Videos S1 and S2.

To more quantitatively measure the mutual influence of the different cells, we measured the number of agents that reached the target (end point), i.e. the success rate in the maze. Failure to reach the target point can result from amoeboid motility that led to an impasse, or from mesenchymal motility with not enough energy to degrade a blocking wall. In this work we are mainly interested in the mutual influence of different cells that migrate in the same environment, and how agents’ individual success varies with changes in the group. The overall success rate, namely the fraction of agents that reached the end zone within the pre-defined simulation time, represents the metastatic ability of the specific cellular phenotype under the given environmental conditions. Although the agents move independently, we refer to the overall success of all agents as the *population* success rate. As the individual success depends on the characteristics of the entire population, the overall success rate is a measure for the fitness of the *population* rather than of a single agent.

**Figure 3:**
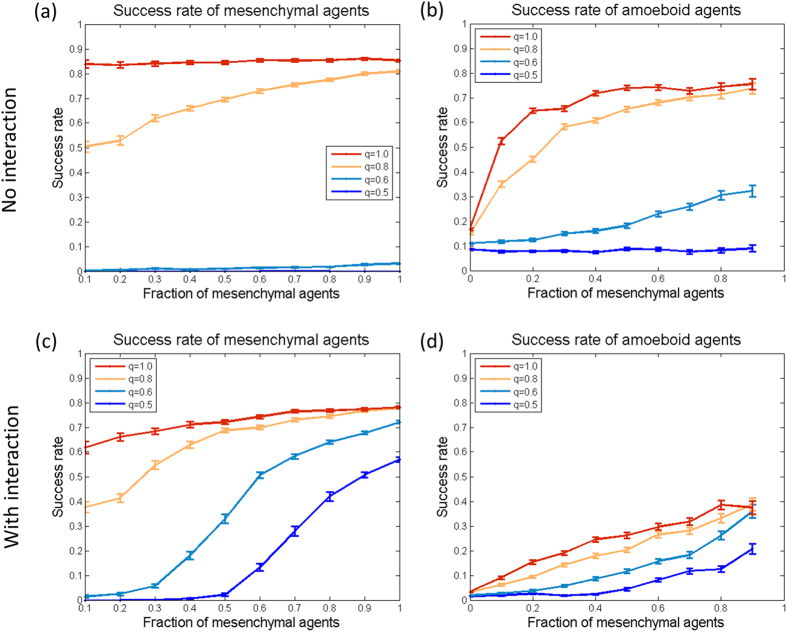
Success rates. We measured the ratio of successful agents in the population, for different mesenchymals/amoeboids ratios and different energy intake rates q = 0.5, 0.6, 0.8, 1.0 from bottom to top. (**a**)-(**b**) With no interaction: the presence of more mesenchymals is beneficial only when the energy is sufficient for effective wall degradation. (**a**) The success rate of mesenchymal agents; (**b**) The success rate of amoeboid agents. (**c**)-(**d**) With constant cell-cell alignment interaction as described in eqs. (7)-(8), 42, with parameter values as indicated in Supporting Table T1. (**c**) The success rate of mesenchymal agents. Interaction improves the success of mesenchymals dramatically for the lower energy levels, but slightly impairs the success rate for high energy. (**d**) The success rate of amoeboid agents. Interaction impairs the success of amoeboids, in all energy states and low mesenchymal population. Interestingly, with interaction and high mesenchymal ratio, the success rate of amoeboids increases due to the efficient path generation.

Figure 3a-b show the success rate for different ratios of mesenchymal/amoeboid agents and for different values of the energy intake rate *q*. Figure 3a shows the success of the mesenchymal agents and Fig. 3b shows the success of the amoeboids. When the energy level is low (*q* = 0.5), the success rate of mesenchymal agents is zero, regardless of their rate in the population. This results from the mesenchymal agents becoming immobilized, as they don’t have enough energy for proteolysis of the maze walls, and their relatively low directional noise does not allow them to bypass the obstacle. The performance of the amoeboids is almost unchanged with the addition of mesenchymal cells, as no new paths are actually created by the blocked mesenchymals. The situation is different when the energy intake rate is higher: the success rate of both the mesenchymals and the amoeboids increases with the addition of more mesenchymal agents. The new paths created by mesenchymal agents are thus used by other mesenchymal as well as amoeboid agents and expedite their journey in the maze.

### The role of Interaction

In a high-density population, cells tend to align and travel in loose groups. This cell-cell interaction can result from cell-cell adhesion or by effective shear forces. Generally speaking, alignment interaction has a typical interaction length, below which the objects reject each other (excluded volume) and above which the interaction vanishes. Since alignment interferes with individual chemotaxis, we investigated the effect it has on population success. To do so, we added repulsion interaction on short distances and alignment interaction on longer distances. The repulsion term has the form:
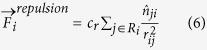
where 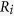 is the set of indices of agents verifying 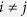 and 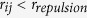, with 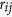 being the distance between agent *i* and agent *j*. 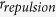 is the repulsion limit, and 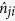 is the unit vector pointing from 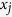 to 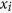 (see Supporting Table T1 for parameter values). Repulsion between close agents simply accounts for their finite volumes and collision avoidance, and is widely used in agent-based models^28^,^34^,^35^.

The alignment interaction is governed by a parameter *w*, which represents the balance between an agent’s direction and the influence of the crowd^35^. The direction vector of an agent is therefore updated according to:
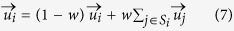
where 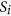 is the set of agents for which 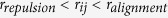 and 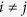. 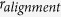 is the maximum range of the alignment interaction. With *dt* being the time step of the numerical integration of eqs. (1)-(7), 27, 11, 15, 21, 27, 36 (see Supporting table T1), the value of 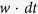 in the simulation was taken to be 0.2, i.e. the crowd has an influence of 20% in determining the new direction (“self confidence” of 80%).

The success rates with interaction, of the mesenchymal as well as amoeboid agents, are shown in Fig. 3c-d. For mesenchymal agents interaction is typically beneficial, especially for the cases of low energy (q = 0.5-0.6) in which interaction rescues the ability of mesenchymal cells to invade and move. As the ratio of mesenchymals is increased, interaction keeps the agents together and allows them to combine forces and degrade the walls together when each agent can’t do it alone. In the high energy levels, however, interaction is insignificant or slightly reduces the success rates. The situation is different for amoeboids: Their success rate declines with the addition of interaction, since grouping impairs their ability to explore the maze. Amoeboid cells are able to move against the dictated external direction, and thus bypass obstacles, by using a relatively large directional noise. Alignment interaction between agents effectively averages the directional noise and significantly interferes with path finding ability.

The abovementioned results suggest that cell-cell interaction should be regulated as a stress-response mechanism for the case of low energy, i.e. metabolic stress. To test such a mechanism we used a dynamic form of *w* as a function of the internal energy *e*. We chose a switch-like function *w(e)*:
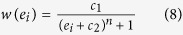
where 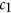, 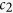 and *n* are constants (see SI Table T1). The function 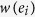 has a value 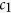 for low values of 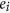 and asymptotically goes to zero as 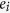 increases.

**Figure 4:**
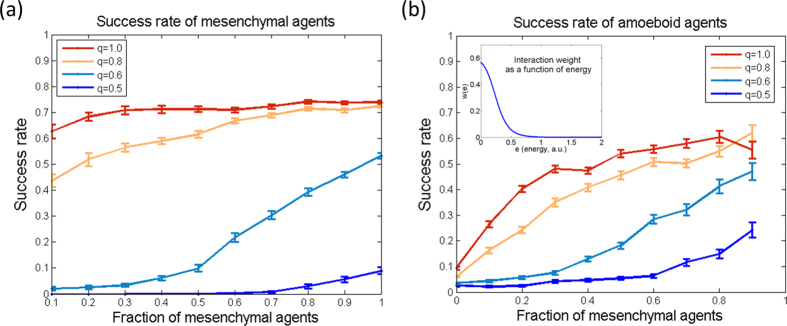
Success rates with dynamic interaction. The success rates of: (**a**) mesenchymal agents; and (**b**) amoeboid agents are shown as a function of the fraction of mesenchymal agents in the population, with dynamic interaction function (inset in (**b**)).

The success rates with dynamic interaction are shown in Fig. 4. The dynamic interaction improves the success rates of both amoeboids and mesenchymals for low energy, and roughly recovers the no-interaction results for high energy (q = 0.8 and above).

We ran our simulation on various mazes, with different topologies, and obtained similar results. While the exact success rates vary between different environments depending on the structure of the maze, the qualitative differences between mesenchymal and amoeboid agents and the role of constant vs. dynamic interaction are virtually unchanged. One other example of different maze results is included in the Supporting Information and Supporting Videos S3 and S4.

### Clustering

**Figure 5:**
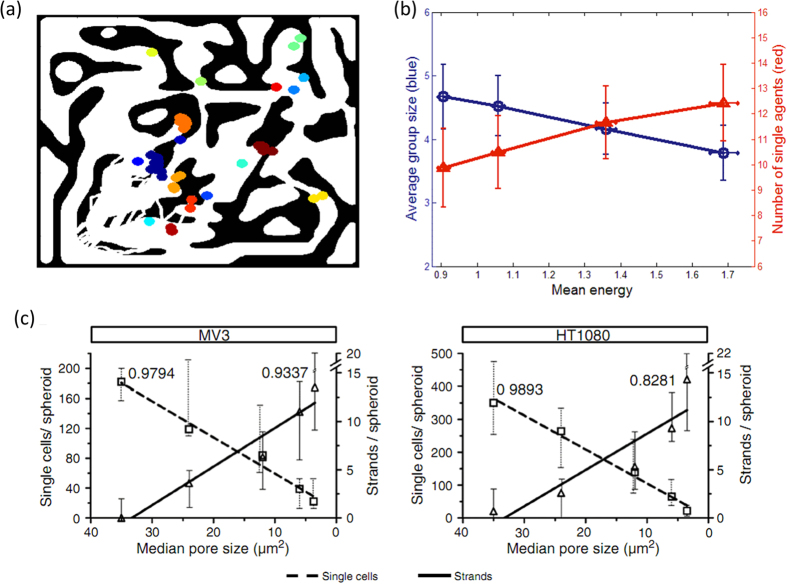
Collective migration and clustering with dynamic alignment interaction. (**a**) A typical snapshot of the simulation, showing agent clustering during migration. Each color marks a separate group. (**b**) Average group size and the number of single agents as a function of the mean energy, for 30% mesenchymals in the population. The group size decreases and the number of single agents increases when the energy is higher. (**c**) Experimental result of Haeger *et al.* (with permission and is licensed from Ref. 19) of melanoma (left) and fibrosarcoma (right) cells migrating in collagen matrix with varying pore size. Increasing stress (smaller pores) induces cellular clustering.

Cell-cell interaction leads to the formation of small groups of cells/agents moving together (Fig. 5a). We measured the average group size and the number of single agents for different energy levels (Fig. 5b). The identification of clusters was done automatically by dendrogram clustering with a defined cutoff radius of 30 (maze size is 800 × 800).

As the average energy increases, the average group size decreases and the number of independently moving agents increases. This is in agreement with experimental data of MV3 (melanoma) and HT1080 (fibrosarcoma) cells moving in collagen matrix with varying pore sizes^19^. In the experiments, small pores were found to induce collective motility (Fig. 5c). Importantly, the mechanical stress induced by the pore size is an equivalent of the metabolic stress inflicted in our model. In the simulation, the ability of an agent to generate a path depends on the ratio between the energy that it has, and the energy that is needed for proteolysis of the wall, which depends on the structure of the wall. Low energy in the simulation means that the cell is unable to cut a sufficient amount of the surrounding walls, and therefore experiences a “stress”. A smaller pore size implies a higher wall mass that needs to be degraded, therefore a higher energy is needed, which effectively scales the available energy to be lower. The formation of clusters in the experiment can be explained by cell-cell adhesion forces that are imposed as a stress-response mechanism.

Measuring cluster sizes for the cases of no interaction and constant interaction does not yield a similar decrease, but a constant level of maximal cluster size (Supporting Fig. S1). Therefore, an energy-dependent interaction is needed in order to obtain a typical behavior which is similar to the experimental results. Other cases of mesenchymal/amoeboid ratios, as well as another maze results, are presented in the SI Text.

## Discussion

Cancer cell migration is a complex process driven by both the cancer cell itself as well as other cells and the surrounding tissue, and involves physical, cellular, and molecular determinants. Cancer invasion is initiated and maintained by signaling pathways that control cytoskeletal dynamics and the turnover of cell-matrix and cell-cell junctions, followed by cell invasion into the tissue. Metastasis then occurs when invading tumor cells engage with blood and lymph vessels, penetrate basement membranes and endothelial walls, and disseminate through the vessel lumen to colonize distant organs^2^,^36^.

In the past decade, intensive cancer research has led to the perception of cancer invasion as a heterogeneous and adaptive process. Cancer cells can migrate as single cells as well as strands and sheets, and can adopt different motility modes, with differences in molecular cytoskeletal dynamics^6^,^11^,^13^,^37^. It is now known that plasticity in cell adhesion, cytoskeletal dynamics and mechano-transduction perpetuates migration and dissemination under varying microenvironmental conditions^38^,^39^.

In a recent work we have shown that high proliferation rate can be advantageous or disadvantageous, depending on the metabolic state, and therefore concluded that co-existence of proliferative and invasive cells may increase the probability for successful metastatic dissemination. In this work we continue to explore the role of tumor plasticity and the coexistence of different cell clones, this time regarding motility mode selection. Upon several stressful environmental conditions, such as MMP inhibitors or hypoxia, EMT cells transform to the amoeboid form, i.e. go through Mesenchymal to Amoeboid Transition (MAT)^7^,^40^. In the population level this transition is gradual, as not all cells transform at the very same time, and mixed populations can be detected at different time points with different ratios of amoeboids and mesenchymals.

In this study we focus on emergent collective effects between adjacent cancer cells. We compare two motility modes, the “path generating” mesenchymal mode used by EMT cells, and the “path finding” amoeboid mode used by EAT/MAT cells, and measure their relative rates of successful invasion in a maze-like environment.

The energy management dynamics in this work is encapsulated in the ratios between the energy intake rate, *q*, and the energy degradation and usage parameters. Obviously, if the intake rate is too small compared to energy demand, the agent will be immobilized. In this work we focused only on the range in which the amoeboid agents are able to move. Mesenchymals, which need more energy for wall degradation, are more vulnerable for energy deficiency. This tuning of the model parameters stems for the experimental observations of MAT (mesenchymal to amoeboid transition) under metabolic stress. For simplicity, we chose to vary *q* alone, but similar results should be obtained by keeping *q* constant and varying the energy usage and degradation parameters. The behavior of the model for different parameter values is discussed in the Supporting Information text and Table 1.

Our computations are done strictly in two spatial dimensions, as most *in vitro* experiments are carried in 2D. Recent experimental advances now provide 3D *in vitro* motility assays, such as implantation of a multi-cellular spheroid in collagen or Matrigel^41^,^42^ or the use of basal membrane crossing using transwells^43^, as well as intra-vital *(in vivo)* microscopy. However, 2D motility can be significant even in allegedly 3D environments, as cells tend to follow muscle fibers, epithelia or wounds^44^, which yield a quasi-2D motility. Our model can be extended to 3D with the proper adjustments and the resulting increase in computational time. However, as we demonstrate here, meaningful qualitative insights can be gained even in a simplified model.

To assess the metastatic potential of a given population, we measured the fraction of agents of both types that managed to arrive at the designated target zone. We found that when the available energy is sufficient, mesenchymal cells typically perform better, as they can move straight ahead using proteolysis. The presence of mesenchymal cells has an advantageous effect on other mesenchymals as well as amoeboids, and the success rate increases with increased EMT population. We therefore predict that under normal conditions (i.e. oxygen and glucose levels) the optimal population should have a mixture of EMT and EAT cells, and a small population of EMT cells should remain as long as MMP is not fully inhibited. New *ex vivo* and *in vivo* experiments are currently being designed and carried to test this prediction.

We studied the effect of multi-cellular invasion by adding alignment interaction between the agents in the model. The interaction force applies to every pair of single agents, but the difference between mesenchymal and amoeboid velocities results in effective interaction between agents of the same type. Multi-cellular migration of EMT cells is beneficial for low energy, and adding interaction rescues the impaired migration if the population of mesenchymals is large enough. We therefore predict that cell-cell adhesion may be induced as a stress-response mechanism, which improves cellular migration and invasion under stress, metabolic or other.

The function of the stress-dependent interaction can be tuned for maximal success rates, depending on the environmental characteristics. However, the qualitative results are robust for a range of the parameters 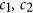 and *n*. In the SI we show the results of another maze with the same interaction function, which yields similar results although the maze is significantly different.

In the experimental work of Haeger *et al.*, the stress factor was the pore size: a smaller pore size resulted in more strands and spheroids and fewer single cells. In other words, a too dense matrix induced cell-cell interaction and adhesion. In our model the stress factor was the energy level, i.e. metabolic stress. Energy shortage in the model results in agents’ disability to degrade the matrix, which is analogous to the dense matrix in the experiment. Our simplified model therefore provides an insight into the mechanism that can account for the transition from single- to multi-cellular migration.

The main conclusion of our work is that both EMT and EAT cells have an important role in successful dissemination, and their path generating and path finding act synergistically to maximize the tumor’s metastatic ability. Therefore, any anti-metastatic treatment should target both types simultaneously, in order for the metastatic spreading to be completely blocked.

## Additional Information

**How to cite this article**: Hecht, I. *et al.* Tumor Invasion Optimization by Mesenchymal-Amoeboid Heterogeneity. *Sci. Rep.* **5**, 10622; doi: 10.1038/srep10622 (2015).

## Supplementary Material

Supporting Information

Supporting Video S1

Supporting Video S2

Supporting Video S3

Supporting Video S4
