# Supplementary material for: Tumor Invasion Optimization by Mesenchymal-Amoeboid Heterogeneity
Source: Sci Rep. 2015 May 27;5:10622. doi: 10.1038/srep10622 (PMC4650638; doi:10.1038/srep10622)
Supplement: Supporting Information [file srep10622-s1.pdf]

Tumor Invasion Optimization by Mesenchymal-Amoeboid Heterogeneity

Inbal Hecht[1#], Yasmin Bar-El[1], Frederic Balmer[2], Sari Natan[3], Ilan Tsarfaty[3], Frank Schweitzer[2] and Eshel Ben-Jacob[1,4#]

[1] School of Physics and Astronomy, The Raymond and Beverly Sackler Faculty of Exact Sciences, Tel Aviv University, Tel Aviv 6997801, Israel
[2] Chair of Systems Design, ETH Zurich, Weinbergstrasse 58, 8092 Zurich, Switzerland
[3] Department of Clinical Microbiology and Immunology, Sackler School of Medicine, Tel Aviv University, Tel Aviv 6997801, Israel.
[4] Center for Theoretical Biological Physics, Rice University, Houston, TX 77005-1827, USA.
[#] Corresponding authors: Inbal Hecht: inbal.hecht@gmail.com; Eshel Ben-Jacob: eshelbj@gmail.com;

I. Supporting Table T1 - model parameters

Parameter	Description	Value
N_a	Number of amoeboid agents	Depends on simulation
N_m	Number of mesenchymal agents	Depends on simulation
N	Total number of agents	50
q	Rate of energy intake (constant in space and time).	Between 0.1 and 1.0
δ	Decay constant of the internal energy	0.3
ζ	Constant characterizing energy cost of ECM degradation. Higher ζ yields lower available energy and lower success for mesenchymals.	1.0
m	Mass of the agents	1.0
γ_a	Friction constant (amoeboid)<br>Affects the velocity and thus the time to target	0.25
γ_m	Friction constant (mesenchymal)<br>Affects the velocity and thus the time to target	1.0
η	propulsion constant	0.5
T_{ori}	Average duration of the *orientation* step of chemotaxis	200
σ^2_{ori}	Variance of the distribution of T_{ori}	20
T_{loc}	Average duration of the *locomotion* step of chemotaxis<br>Longer T_{loc} results in fewer turns which mostly affects the amoeboids and their success rate	10
σ^2_{loc}	Variance of the distribution of T_{loc}	2
$\sigma^2_{compass\_amb}$	Compass noise variance of amoeboids (zero mean)<br>Higher values result in a more jagged trajectory, and better "path finding" ability	0.2 * π
$\sigma^2_{compass\_mes}$	Compass noise variance of mesenchymals (zero mean). Higher values result in a more jagged trajectory	0.15 * π
t_{max}	Simulation time	2500
dt	Time step	0.1
c_r	Repulsion coupling constant	25
$r_{repulsion}$	Repulsion cutoff distance	3
w	Weight of constant alignment interaction<br>w represents the influence of the crowd, compared to "self-confidence" (1-w).	0.2 * dt
$r_{alignment}$	Alignment cutoff distance	6
c_1	Dynamic interaction coefficient (amplitude)	0.6
c_2	Dynamic interaction coefficient (offset)	0.75
n	Dynamic interaction coefficient (sharpness)	10

II. Additional data and results

In Fig.5 In the main text we present the average group size and the number of single agents for the case of 30% mesenchymals and dynamic interaction. The simulation in Fig. 5 was run using the maze presented in Fig. 2 and denoted “Maze A” hereafter. Fig. S1 below compares group sizes for the different interaction forms: no interaction, constant interaction and dynamic interaction. Only in the case of dynamic interaction, the behavior is similar to the experimental results (ref. [19] in the main text), while with constant interaction or no interaction, the group size and number of single cells are constant and do not depend on the energy. Similar results are obtained for 10% mesenchymals, as shown in Fig. S2.

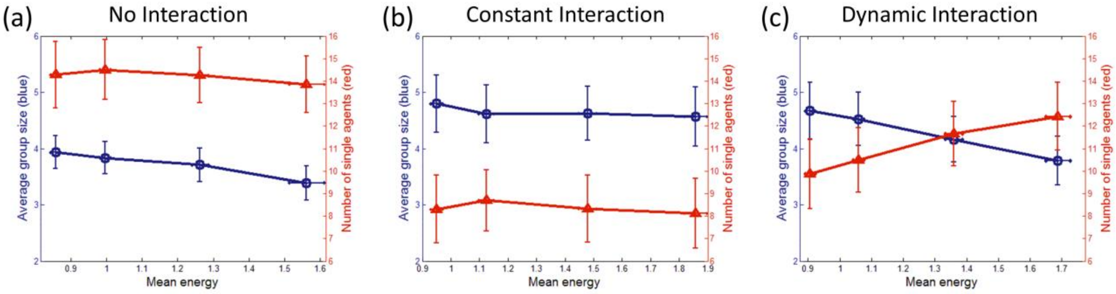

Fig. S1. Comparison of group size and number of single agents in Maze A, between the cases of no interaction (a), constant interaction (b) and dynamic interaction (c) for the case of 30% mesenchymals.

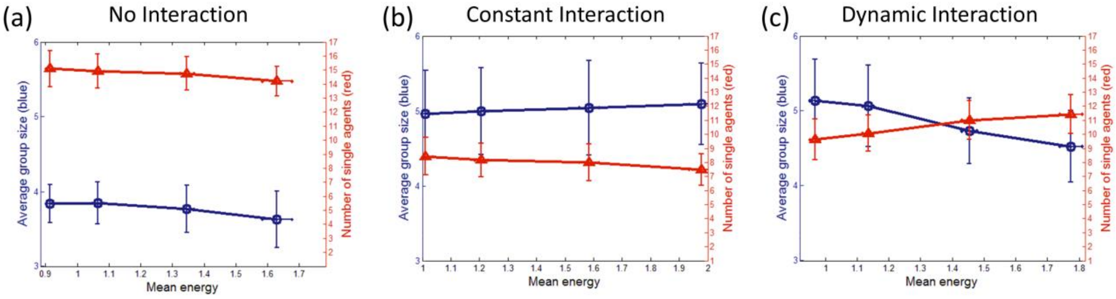

Fig. S2. Comparison of group size and number of single agents in Maze A, between the cases of no interaction (a), constant interaction (b) and dynamic interaction (c) for the case of 10% mesenchymals.

The situation is slightly different for 80% mesenchymals, as can be seen in Fig. S3. Mesenchymal cells move in a relatively straight path, and therefore when there are many mesenchymal cells, their motion resembles that of a cluster. This is especially prominent when the energy is low, and the cells get stuck or move very slowly. This is the reason for the large group size even without interaction, and the decrease in group size as the energy increases.

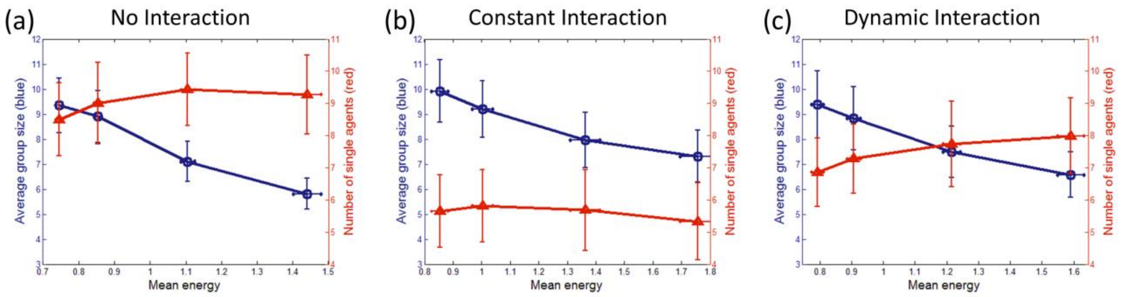

Fig. S3. Comparison of group size and number of single agents in Maze A, between the cases of no interaction (a), constant interaction (b) and dynamic interaction (c) for the case of 80% mesenchymals.

III. A different maze – Maze B

In addition to the results presented in the main text, we have tested several other mazes with different topographies and densities. In all cases the qualitative results were similar. Here we present detailed results for one additional maze, marked "Maze B" in the subsequent results. The maze is presented in Fig. S4a-b, with some typical trajectories for mesenchymals and amoeboids, respectively.

The success rates in Maze B are shown in Fig. S5, with no interaction, constant interaction and dynamic interaction. The results are qualitatively similar to the results of Maze A in the main text (Figs. 3-4), with basal higher success.

With the added interaction, the agents/cells tend to move in groups, as demonstrated in Fig. S6 for Maze B (see also Fig. 5 in the main text for the original maze). We measured the average group size and the number of single agents in this maze as well, for different energy levels (See also Fig. 5 in the main text). The results, for different amoeboid/mesenchymal ratios, are presented in Figs. S7-S9. For 10% and 30% mesenchymals and dynamic interaction we obtain a decrease in the group size and an increase in the number of single agents as the energy increases, similarly to the experimental results of Haeger et al. (Fig. 5c-d in the main text and ref. [19] therein). This behavior is not obtained for the cases of no interaction or constant interaction. However, with 80% mesenchymals the behavior is slightly different, as the agents move very closely when the energy is low, even without interaction. These results are very similar to the results with Maze A (Figs. S1-S3 above).

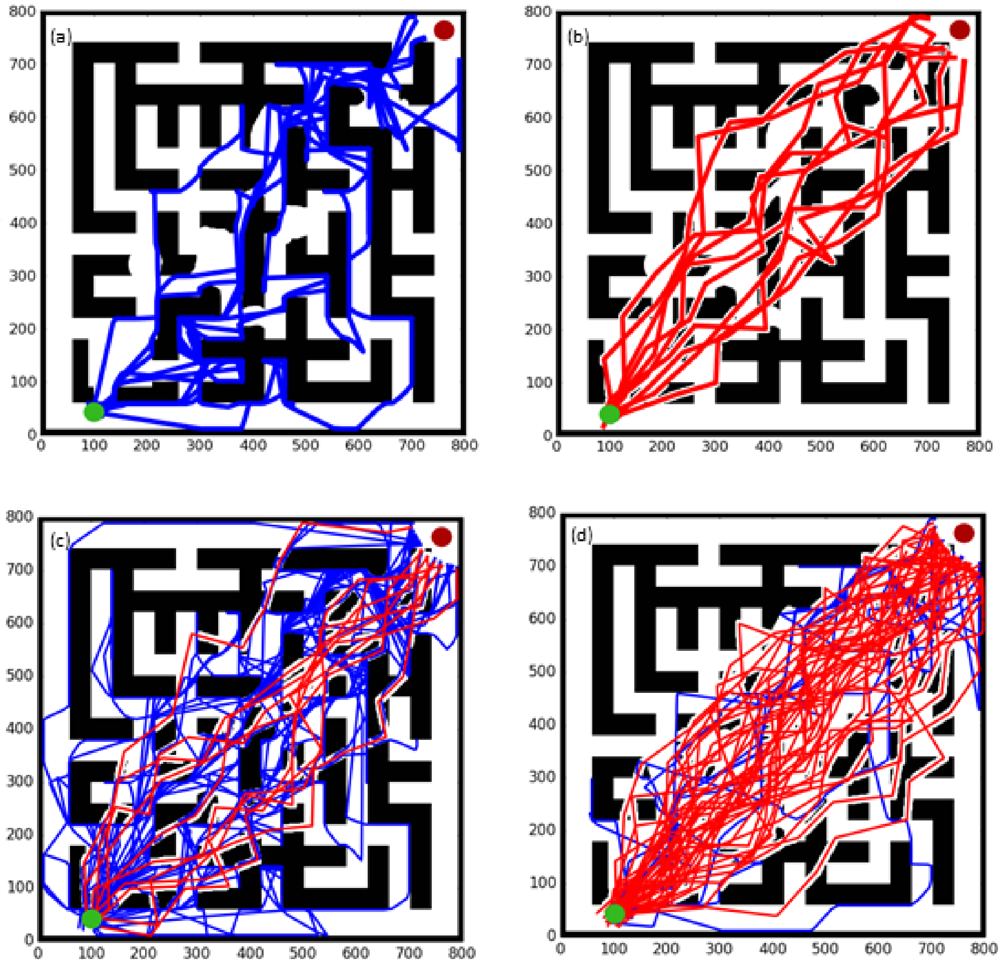

Fig. S4. An example of a different maze – Maze B. The maze with trajectories for (a) 10 amoeboids; (b) 10 mesenchymals; (c) 20% mesenchymals and 80% amoeboids (total 50 agents); (d) 80% mesenchymals and 20% amoeboids (total 50 agents). The starting point is marked in green and the target (end) point is marked in dark red.

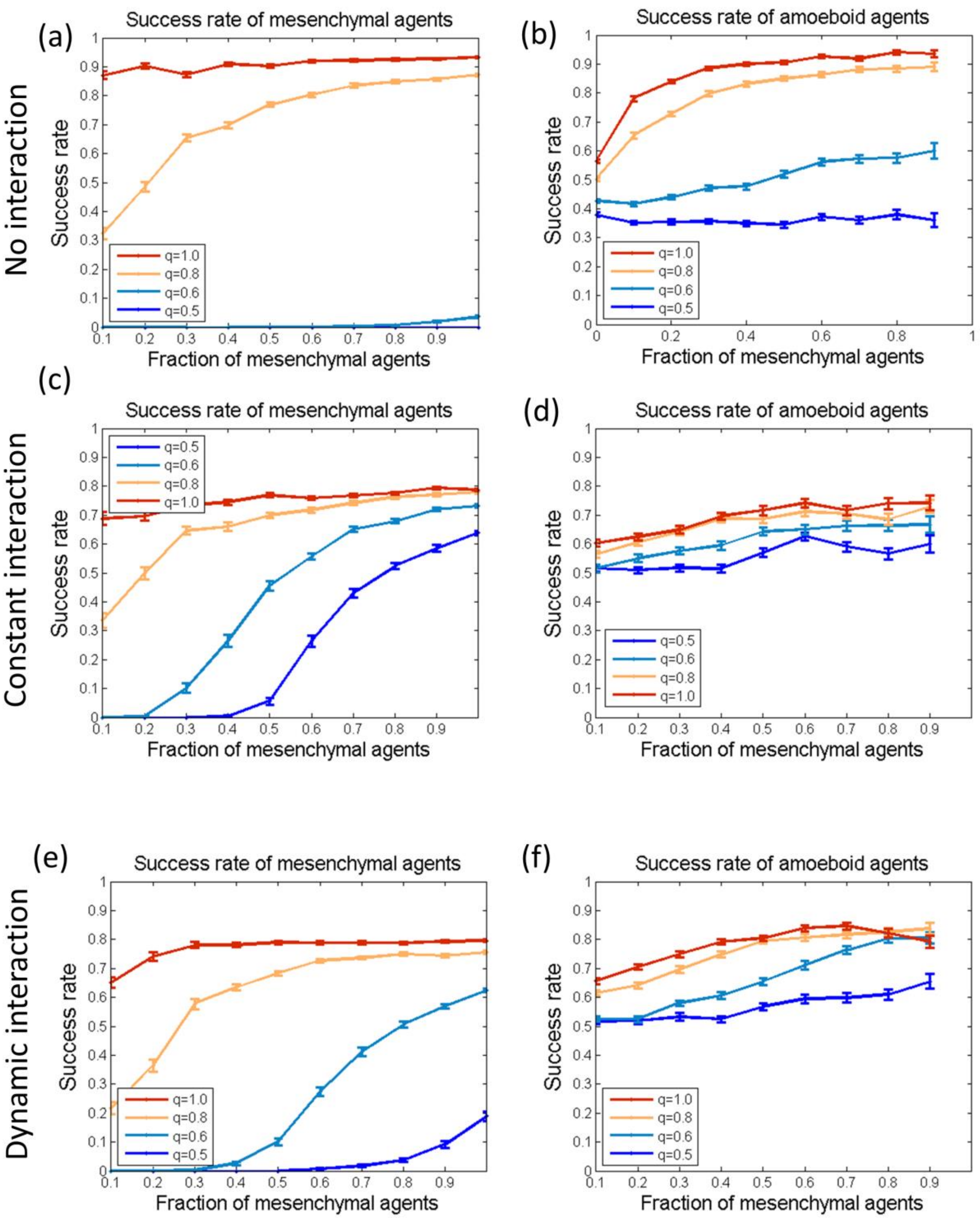

Fig. S5. Success rates for amoeboid and mesenchymal agents in Maze B. (a)-(b) no interaction; (c)-(d) constant interaction; (e)-(f) dynamic interaction (see the main text, eq. 9).

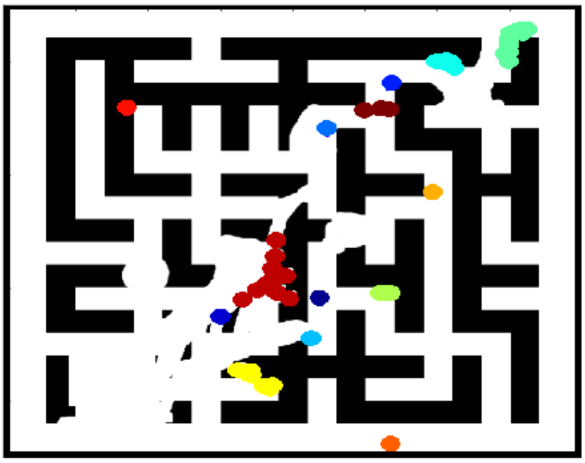

Fig. S6. Clustering in Maze B. A simulation snapshot showing cellular groups for Maze B, q=0.6 and 30% mesenchymals. Groups are identified using the dendrogram algorithm, based on cell-cell distances. Each group is marked by a different color.

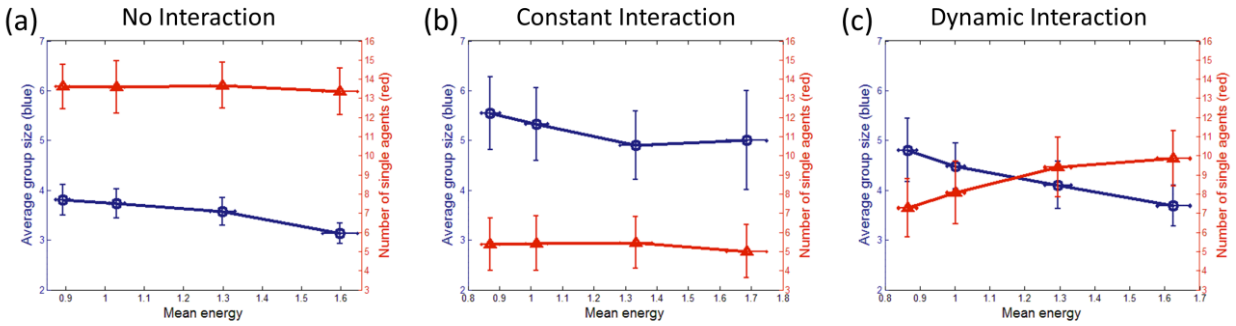

Fig. S7. Clustering in maze B, 30% mesenchymals. The average group size (blue, left axis) and the number of single agents (red, right axis) are shown for: (a) no interaction; (b) constant interaction; (c) dynamic interaction. Only a stress-dependent interaction results in a qualitative behavior that is similar to real biological results (Fig. 5 in the main text).

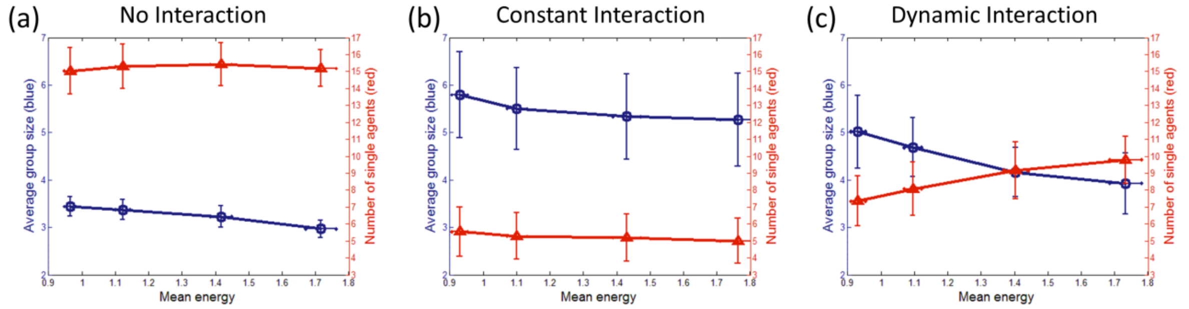

Fig. S8. Clustering in the new maze, 10% mesenchymals. The average group size (blue, left axis) and the number of single agents (red, right axis) are shown for: (a) no interaction; (b) constant interaction; (c) dynamic interaction. Only a stress-dependent interaction results in a qualitative behavior that is similar to real biological results (Fig. 5 in the main text).

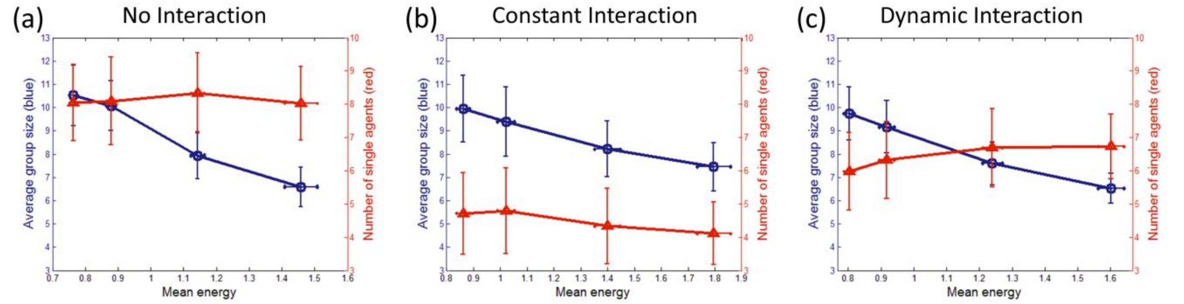

Fig. S9. Clustering in the new maze, 80% mesenchymals. The average group size (blue, left axis) and the number of single agents (red, right axis) are shown for: (a) no interaction; (b) constant interaction; (c) dynamic interaction. Only a stress-dependent interaction results in a qualitative behavior that is similar to real biological results (Fig. 5 in the main text).

IV. Supporting videos

Movie S1. Maze A (as presented in the main text) with q=1.0 and 20% mesenchymals.

Movie S2. Maze A (as presented in the main text) with q=1.0 and 80% mesenchymals.

Movie S3. Maze B (as presented in the SI text) with q=1.0 and 20% mesenchymals.

Movie S4. Maze B (as presented in the SI text) with q=1.0 and 80% mesenchymals.
